# Latent class analysis was accurate but sensitive in data simulations^☆^

**DOI:** 10.1016/j.jclinepi.2014.05.005

**Published:** 2014-10

**Authors:** Michael J. Green

**Affiliations:** MRC/CSO Social and Public Health Sciences Unit, University of Glasgow, 200 Renfield Street, Glasgow, G2 3QB, United Kingdom

**Keywords:** Latent class analysis, Development, Longitudinal, Trajectories, Heterogeneity, Simulations

## Abstract

**Objectives:**

Latent class methods are increasingly being used in analysis of developmental trajectories. A recent simulation study by Twisk and Hoekstra (2012) suggested caution in use of these methods because they failed to accurately identify developmental patterns that had been artificially imposed on a real data set. This article tests whether existing developmental patterns within the data set used might have obscured the imposed patterns.

**Study Design and Setting:**

Data were simulated to match the latent class pattern in the previous article, but with varying levels of randomly generated variance, rather than variance carried over from a real data set. Latent class analysis (LCA) was then used to see if the latent class structure could be accurately identified.

**Results:**

LCA performed very well at identifying the simulated latent class structure, even when the level of variance was similar to that reported in the previous study, although misclassification began to be more problematic with considerably higher levels of variance.

**Conclusion:**

The failure of LCA to replicate the imposed patterns in the previous study may have been because it was sensitive enough to detect residual patterns of population heterogeneity within the altered data. LCA performs well at classifying developmental trajectories.

## 1 Introduction

**What is new?**

• Latent class analysis can be a useful tool for classifying developmental trajectories.
• The caution advised previously in the use of this method may have been overstated.
• Using only the Bayesian Information Criterion to determine the number of classes may in some circumstances result in more classes than are substantively useful.

As longitudinal data from prospective cohort studies have proliferated, there has been a growing interest in distinguishing between different developmental trajectories. This can be done to provide a description of development within a population or to study the consequences or predictors of particular patterns of development [1]. A number of different statistical methods are available, which have the general purpose of classifying individuals into heterogeneous groups with homogeneous developmental trajectories (ie, where those within a group are very similar to one another, but the groups are very different from each other) [1], [2], [3]. These include a number of methods based on structural equation modeling such as latent class analysis (LCA) [3], latent class growth analysis (LCGA) [2], and latent class growth mixture modeling (LCGMM) [2]. I will refer to these collectively as latent class methods. A recent study in this journal by Twisk and Hoekstra (T&H) [1] examined how well these methods perform, concluding that “great caution” was needed in their application as latent class methods did not perform well at identifying developmental trajectories or at classifying individuals, particularly where there were nonlinear trajectories. The T&H study addressed an important question and makes several valuable points about the comparative utility of these methods, but the need for caution may have been overstated.

**Fig. 1:**
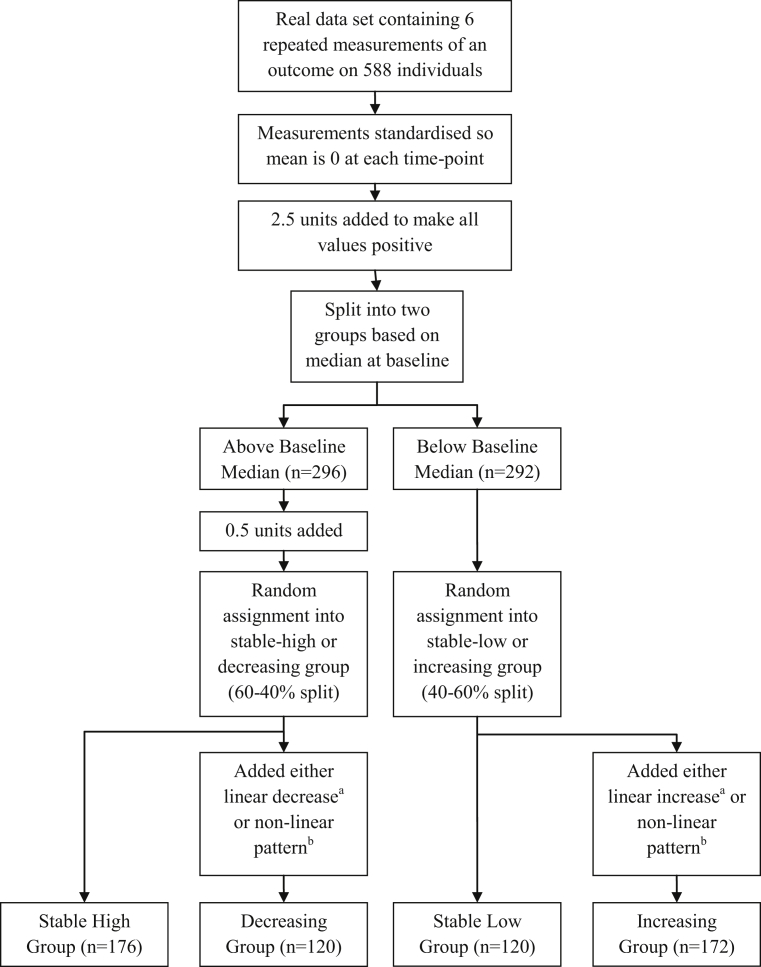
Flowchart describing simulation of data by Twisk and Hoekstra. ^a^0.5 units per time point. ^b^1 unit change per time point with direction of change reversed at fourth time point.

The simulated data used by T&H [1] were created by starting with real data from 588 individuals, measured on six separate occasions and then altering these data to impose a latent class structure. The altered data may have retained some of the original population heterogeneity, which might, if detected by the latent class methods, have obscured the imposed or simulated heterogeneity. The steps taken by T&H to manipulate the data are described in Fig. 1. The first step was to standardize the measurements at all time points so that the average developmental trajectory would have been flat at the mean of 0. It is worth considering what might have occurred if latent class methods were applied to the data in this state, without any further manipulation. Although the average trajectory had been modified to 0, there may still have been subgroups of individuals with particular patterns of deviation from this trajectory, for example, with measurements consistently above or below the mean. Whether this population heterogeneity would have been detectable using latent class methods would only be possible to ascertain using the original data. Further data manipulations were then applied to create four latent classes: one with stable high values, one with stable low values, one with an increasing linear trend, and one with a decreasing linear trend. Importantly, these further manipulations altered only the mean values within each class, while retaining the original population variance. Assignment using the median from the first measurement (third step in Fig. 1) means that the population heterogeneity might not have been randomly distributed across the four classes. The latent class methods were then tested by applying them to the altered data and seeing whether they could identify the imposed structure and correctly classify individuals within it. Such a test assumes that any retained population heterogeneity within the altered data would have been negligible relative to the imposed heterogeneity (or imposed mean structure). If however the population heterogeneity was not small relative to the imposed heterogeneity, it could have been detected by the latent class methods, obscuring the imposed patterns and meaning the latent class methods would appear to fail the test. To demonstrate this point, some further simulations are reported here, which recreate this imposed mean structure while controlling the amount of additional variance (or heterogeneity) around that structure. It is hypothesized that when the additional variance is low, the imposed heterogeneity will be identifiable, whereas high levels of additional variance will make it harder to detect.

## 2 Methods

### 2.1 Data

Six simulated data sets were created using SPSS version 19.0 and were based on the imposed latent class structures in the article by T&H [1]. Each data set therefore contained four classes with respective *n* of 120, 172, 176, and 120, summing to a total of 588 cases. Data were generated randomly based on a normal distribution around the mean values within each class for each of the six measurements (T1–T6) as reported in the online appendices of the article by T&H [1]. Thus, in contrast to the previous simulations, any additional variance beyond the imposed latent class structure will be entirely random. Standard deviations for the normal distribution were also taken from the appendices of the article by T&H [1], which meant that the variances differed across the four classes. To control the amount of additional variance around the latent class structure, three data sets were created, representing three conditions: similar variance (using the standard deviations as reported), lower variance (using the standard deviations divided by two), and higher variance (using the standard deviations multiplied by two). This procedure was repeated for both the linear and nonlinear latent class structure. Only one data set was simulated for each set of conditions as this was considered sufficient to demonstrate the point, and those interested in more rigorous LCA simulations using multiple data sets are referred to the existing literature [4], [5], [6].

### 2.2 Analysis

LCA was performed on each of the simulated data sets using Mplus 7 [7], and the results were compared with the original mean values and class memberships. LCA rather than LCGA or LCGMM was chosen for pragmatic reasons as I was familiar with it from prior use [8], [9], and one method was considered sufficient to demonstrate the point about residual population heterogeneity, which would apply whichever method was used. Whereas, LCGA and LCGMM use intercept and slope parameters from repeated observations as indicators of latent classes, LCA uses the observations themselves as indicators. This means there is no assumption of any particular linear form (ie, quadratic, cubic, and so forth). The LCA model for continuous data is described in detail elsewhere [4], [6] and assumes that observations are independent conditional on class. LCA solutions were arrived at by taking the best-fitting solution from 100 random sets of starting values. The number of latent classes in LCA is usually determined by comparison of models with different numbers of classes along model fit criteria such as the Bayesian Information Criterion (BIC) [10]. For comparability, the four-class LCA solutions are reported, despite additional classes tending to produce further improvements in the BIC.

## 3 Results

**Fig. 2:**
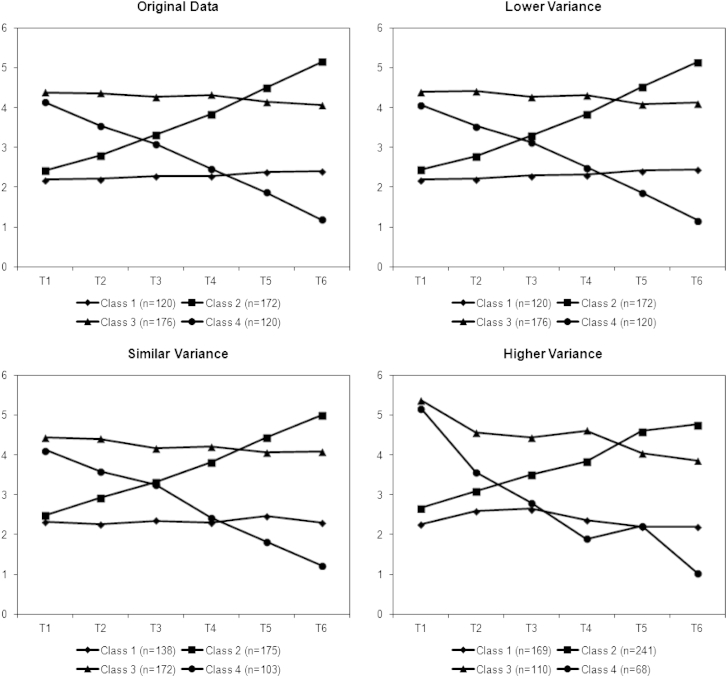
Mean estimates from latent class analysis simulations of linear classes and original mean values.

**Fig. 3:**
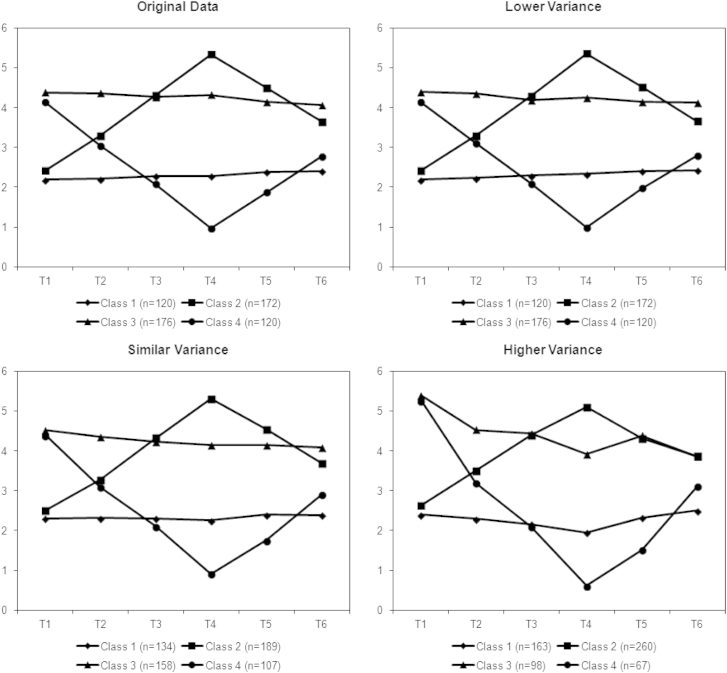
Mean estimates from latent class analysis simulations of nonlinear classes and original mean values.

Fig. 2 shows the mean estimates from the linear data sets compared against the original mean values, and Fig. 3 shows those from the nonlinear data sets. LCA seemed to perform well at reproducing the mean values for each class from the original data in both the lower and similar variance conditions. Only in the higher variance condition did the estimates begin to differ, and even these were still similar to the original means.

Table 1 shows for each data set the proportion of individuals correctly classified within their original class. Results were similar for both the linear and nonlinear data sets. Classification was perfectly accurate to the original classes in the lower variance condition, and there was only a small amount of misclassification in the similar variance condition (∼5%). In the higher variance condition, approximately a quarter of the population was misclassified.

**Table 1:** Proportion within each simulated data set correctly classified to their original class

Variance condition	Linear trends	Nonlinear trends
	Percent correctly classified (*n* = 588)	Percent correctly classified (*n* = 588)
Lower variance	100.0	100.0
Similar variance	95.9	94.7
Higher variance	72.6	75.2

**Fig. 4:**
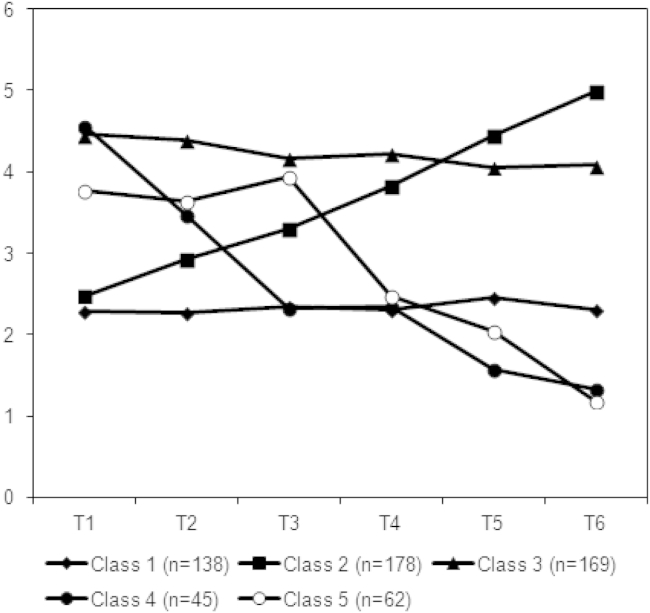
Five-class solution from linear, similar variance condition.

In the higher variance conditions, the minimum BIC criterion selected five and seven classes respectively for the linear and nonlinear models as the optimal solutions. In the lower and similar variance conditions, the BIC continued to improve to between 8 and 10 classes at which point 100 random sets of starting values no longer produced a replicable solution (a point returned to later). To demonstrate what happened when additional classes were included, Fig. 4 shows a five-class solution from the linear, similar variance condition. Introducing an additional class appears to split the decreasing class into two further groups with very similar trajectories, differing mainly in terms of a high or low value at T3.

## 4 Discussion

In this simple simulation, LCA performed quite adequately even with similar levels of variance to those used previously [1] where a latent class structure was not accurately reproduced. The main difference between the data used here and those used previously was in the nature of the additional variance around the imposed latent class structure. The additional variance in this article was purely random and based on a normal distribution, whereas the additional variance in their article was derived from real population data on repeated measurements of some outcome. It might be argued that starting with real data is more valid or generalizable to a real-life research setting, but in a real research setting, one is attempting to describe the total population heterogeneity without any distinction between imposed and additional variance. Starting with real data, it is more likely that the variance would retain residual heterogeneous patterns that could have obscured the imposed heterogeneity. This suggests that LCA did not fail to reproduce the imposed classes because it is an inaccurate method, but because it was sensitive enough to detect the residual population heterogeneity within their data. LCA can therefore still be considered a powerful and useful tool for classifying and analyzing heterogeneous developmental trajectories.

Manipulating the amount of random variance around the imposed latent class structure did demonstrate difficulties in classifying respondents when the random variance was high. This may be because LCA was sensitive enough to detect distinct patterns within the random variance, but it does highlight that LCA may classify individuals more definitively in some settings than others, so for example, entropy statistics [11] should be considered and reported and, as T&H point out [1], the uncertainty in latent class assignments should be taken account of in further analyses [12]. Additionally, the LCA model assumes conditional independence of observations within class, that is, it is assumed that all the autocorrelation between repeated observations is captured by the class structure. This may not always be desired when modeling longitudinal growth or development, as one might wish to allow for some autocorrelation of observations within a broad classification system. In such cases, more sophisticated methods such as LCGMM may be more appropriate, although as T&H indicate, this comes at the price of increased computational complexity [1].

Previous simulation studies have indicated that the BIC gives either an accurate or a low estimate of the number of classes within a data set [4], [5], [6]. T&H [1] found however that when the number of latent classes was determined empirically using the BIC, LCA and LCGA tended to identify a higher number of classes than the four imposed, and this finding was replicated here; modeling additional classes led to further reductions in the BIC. Further simulation studies might investigate whether there are particular conditions under which this occurs. If so, this may again be because LCA is sensitive rather than inaccurate. It has been noted previously that a continuous distribution can be approximated by a discrete distribution [13]. Even in a situation with random variation around a single flat trajectory, chance alone could result in some distinct patterns emerging, for example, the five-class model in Fig. 4 showed two decreasing trajectories which differed in terms of the measurement at T3. It appears that LCA is sensitive enough to detect such patterns of variation over and above an imposed latent class structure. If one's objective with LCA is purely descriptive, this could be seen as an advantage, providing a detailed and sensitive description of population heterogeneity. However, such sensitivity could be considered a disadvantage in research aimed at studying the predictors or outcomes of particular trajectories. In such cases, one generally wants the lowest number of classes that can adequately describe the heterogeneity [2], [9], as additional classes can complicate further analyses, with interpretation being especially difficult where there is little substantive or meaningful difference between two classes. Collins and Lanza [3] point out that statistically significant differences between two classes may have little impact on their substantive interpretation and that in such cases, it may be advisable to use a more parsimonious solution with fewer classes. The difference at T3 between classes 4 and 5 in Fig. 4 for example might only be important if it was clinically meaningful. Also where an additional class only contains a small number of individuals, it may not be a useful distinction as the small numbers will make it difficult to say anything meaningful about associations with other variables. Thus, optimizing the BIC may not always be the best method for selecting the number of latent classes; one might need to think more theoretically about how the classes will be interpreted.

It should be noted that this article has dealt with LCA only as applied to continuous data not categorical data. LCA, relative to LCGA or LCGMM, may be particularly useful for describing development over time on categorical measures, as it deals more easily with nonlinear patterns, requiring no prespecified assumptions about linear form [3]. Categorizing continuous data along clinically meaningful thresholds could also be a sensible response to the sensitivity of LCA and could help ensure that additional classes do represent clinically meaningful distinctions. This article has also dealt mainly with identification of classes and classification of individuals into those classes. Other simulation studies have examined issues around relating class membership to covariates in subsequent analysis [12], [14].

Finally, T&H point out that “classifying developmental trajectories is mostly not the only solution to answer certain research questions” [1]. This is true and other methods may well perform better in certain situations, but latent class methods should not be dismissed as they can be effective, useful, and relatively intuitive. For example, T&H suggest using individual growth parameters as predictors of a future outcome [1], but where growth is not linear, this will mean at least three individual parameters (an intercept, linear slope, and quadratic slope), and the contributions of these parameters could be difficult to interpret, especially where they are found to interact. Such a situation becomes more complex still if one wishes to include parameters from more than one growth curve to represent development across multiple domains, whereas this would be handled relatively easily using latent class methods.

Overall, it appears that LCA can accurately classify individuals to distinct developmental trajectories, but that in some circumstances, it can be sensitive enough to detect a higher number of distinct groups than may be useful for subsequent analysis.
